# HIV-related knowledge in Nigeria: a 2003-2013 trend analysis

**DOI:** 10.1186/s13690-018-0268-2

**Published:** 2018-04-23

**Authors:** Lena Faust, Michael Ekholuenetale, Sanni Yaya

**Affiliations:** 1 Faculty of Health Sciences, University of Ottawa, Ottawa, ON Canada; 2 Department of Epidemiology and Medical Statistics, Faculty of Public Health, College of Medicine, University of Ibadan, Ibadan, Nigeria; 3 School of International Development and Global Studies, University of Ottawa120 University Private, Ottawa, ON K1N 6N5 Canada

**Keywords:** HIV, HIV-related knowledge, Trend analysis, Nigeria, Demographic and health survey

## Abstract

**Background:**

Given Nigeria’s status as the country with the second highest number of people living with HIV globally, and 9% of the total global burden of HIV being attributable to Nigeria alone in 2013, improving our understanding of the nature of the HIV epidemic in Nigeria is crucial. As HIV-related knowledge may be an important contributor to engagement in preventive behaviours, it is of interest to investigate trends in HIV-related knowledge in Nigeria with the purpose of informing future HIV prevention and education efforts. This study therefore aims to investigate trends in HIV-related knowledge in Nigeria between 2003 and 2013.

**Methods:**

Data were derived from the 2003-2013 Nigerian Demographic and Health Surveys, and HIV-related knowledge scores were computed based on answers to HIV-related knowledge questions in the surveys. The significance of the difference between HIV-related knowledge across the time points was determined via the Kruskal-Wallis test, and changes in HIV-related knowledge were displayed graphically, stratified by relevant socio-demographic characteristics. ARIMA models were fit to the 2003 to 2013 trend data.

**Results:**

Although there was generally a decrease in HIV-related knowledge across most knowledge domains in 2008, an overall increase was observed between 2003 and 2013. Unfortunately however, this was not the case for knowledge of mother-to-child transmission, which decreased between 2003 and 2013. The disparity in knowledge of HIV risk reduction between states also increased over time.

**Conclusion:**

These findings suggest that although HIV-related knowledge appears to be increasing overall, future HIV prevention and education programs should focus on specific knowledge domains such as mother-to-child transmission, and on specific states in which HIV-related knowledge remains low.

## Background

As of 2015, 3.5 million people were estimated to be living with HIV in Nigeria, and the disease is estimated to have resulted in 180,000 deaths in that year [1]. Given Nigeria’s status as the country with the second highest number of people living with HIV globally, and 9% of the total global burden of HIV being attributable to Nigeria alone in 2013 [2], improving our understanding of the nature of the HIV epidemic in Nigeria is crucial.

Previous studies have emphasised that increasing HIV-related knowledge is a critical aspect of HIV prevention [3–5], underlining its influence on the likelihood of engagement in preventive behaviours [6], but few studies have examined trends in HIV-related knowledge in Sub-Saharan Africa, with one study doing so in Uganda [7], and others in Ethiopia [8] and South Africa [9]. Importantly, despite the fact that low HIV-related knowledge, and consequently lower engagement in preventive behaviours, is considered a relevant factor in the transmission of HIV in Nigeria, HIV-related knowledge levels in the country have not been studied in detail [10]. Although a prior study in Nigeria examines the association of HIV transmission and prevention knowledge indicators with HIV-related stigmatization [11], trends in these knowledge indicators themselves were not investigated, and these remain to be analysed, particularly in the context of national efforts to improve awareness and knowledge of HIV.

In working towards the millennium development goal of halting and beginning to reverse the spread of HIV/AIDS by 2015, Nigeria launched the National Strategic Plan (NSP) to combat HIV/AIDS. This program ran from 2010 to 2015, and focused on prevention, aiming to reduce the transmission of the disease through the modification of behavioural practices and improving public HIV-related knowledge [10]. Importantly however, despite including the support of research activities and the reduction of gender inequities in its mandate, Nigeria’s National Agency for the Control of AIDS (NACA) reports that evidence-based programing and gender-based approaches in the strategy remain to be improved [10]. As such, this study will investigate the trends in HIV-related knowledge between 2003 and 2013, and stratify the investigation of these trends by socio-demographic characteristics such as gender and income, in order to determine whether HIV-related knowledge has increased since the implementation of the NSP, and whether this increase differs among socio-demographic groups. The selection of these socio-demographic factors is based on a recent study by the authors, in which logistic regression analyses indicated that factors including poverty, low literacy, and being female, among other factors, are associated with a higher likelihood of having low HIV-related knowledge [12].

As prior studies in Sub-Saharan Africa have reported that wealth inequality, rather than absolute wealth or poverty, is a stronger driver of HIV transmission [13–16], and a prior study using Nigerian data has found that, particularly among females, high wealth inequality is associated with lower HIV-related knowledge [12], the observed trends in HIV-related knowledge over time will also be described with respect to state-level wealth inequality rather than solely absolute wealth. Investigating changes in the levels of HIV-related knowledge prior to and following the implementation of the NSP, and observing differences in these changes across various socio-demographic strata will shed light on whether or not the NSP was effective in increasing general population-level knowledge and understanding of HIV transmission, and in which socio-demographic groups this was most or least successful, in order to inform future national HIV education efforts and more specific targeting of such efforts among priority groups.

### Study objective

The purpose of this study was to describe trends in HIV-related knowledge in Nigeria from 2003 to 2013.

## Methods

### Data source

This study is based on the 2003, 2008, and 2013 Nigerian Demographic and Health Surveys (NDHS) [17–19], nationally representative surveys of men and women in Nigeria. As the 2013 survey only contained data on respondents aged 15 to 49 years, cases older than 49 years in the 2003 and 2008 datasets were excluded from this analysis, giving total final sample sizes of *n* = 9713, *n* = 47,193, and *n* = 56,307 for 2003, 2008, and 2013, respectively. As the survey is based on females in all of the sampled households and males in half of the sampled households (in 2008 and 2013) or one third of the sampled households (in 2003), there is greater representation of females than males in these surveys. The sampling procedure for the NDHS involved a 3-stage (in 2013) or 2-stage (in 2003 and 2008) stratification, in which respondents were first stratified by urban versus rural dwelling, and households were subsequently selected using equal probability sampling. This sampling method was taken into account in the computation of survey weights, applied to ensure the representativeness of the sample with regards to the general population. Data for this study is derived from the individual female and male datasets in each year, merged prior to data analysis.

### Variables measured

The outcome variable, HIV-related knowledge, was computed as the sum of correct answers to HIV-related awareness and knowledge questions in the NDHS. For questions assessing HIV-related knowledge, answers were recoded as follows: correct answer = 1, incorrect answer = 0, do not know = 0 (see Appendix). For questions assessing HIV-related awareness (questions 1-3, Appendix), aware = 1, unaware = 0. Twelve questions were included in the HIV-related knowledge total score (shown in Appendix), giving a highest possible total score of 12. For a more detailed analysis of different areas of HIV-related knowledge, these 12 questions were then also separated into four knowledge domains (general HIV-related knowledge, knowledge of risk reduction measures, general knowledge of transmission routes, and knowledge of mother-to-child transmission), with a highest possible score of 3 in each category.

Although the main independent variable is time, changes in HIV-related knowledge over time were analysed stratified by several socioeconomic and demographic factors, identified as predictors of HIV-related knowledge in a recent paper [12]. These factors included age, sex, rural or urban residency, literacy level, educational attainment, employment status, ethnicity, religion, absolute wealth, and state-level wealth inequality.

Literacy was recoded from its initial categories into dichotomous categories (literate vs. low literacy level/illiterate/visually impaired), and cases for which a literacy assessment card was unavailable were coded as missing and dropped from subsequent analyses. Absolute wealth was defined using the continuous wealth scores calculated in the NDHS, which are derived from an asset index of household goods (such as the ownership of livestock), and subsequently categorized into quintiles at the national level. State-level wealth inequality was calculated through an additive transformation of the continuous wealth scores to give only positive values [14], followed by the sorting of transformed scores by state, and the computation of the ratio of the lower over the upper wealth quintile to produce a state-level wealth inequality ratio.

### Data analysis

As the distributions of scores for all four HIV-related knowledge domains as well as the overall HIV-related knowledge score were non-normal (3SD > mean) in one or more of the three years, analysis of variance (ANOVA) was not suitable, and thus the Kruskal-Wallis test was applied to determine whether these distributions differed significantly across the three years. The changes in mean scores for each knowledge domain and overall HIV-related knowledge from 2003 to 2013 were then displayed graphically, stratified by the relevant aforementioned socioeconomic and demographic variables. Autoregressive Integrated Moving Average (ARIMA) models were fit to the 2003-2013 trend in HIV-related knowledge for all knowledge domains. Possible predictors were selected for the models based on a prior analysis of socio-demographic determinants of HIV-related knowledge in a forthcoming paper, and were entered into the models individually. The ARIMA models with the most accurate fit (those resulting in the highest R-squared value and lowest mean absolute percentage error (MAPE)) are presented.

### Ethics approval

The DHS program obtains informed consent from participants prior to data collection, and all data is anonymous, with survey respondents remaining unidentified. Ethics approval for all surveys carried out by the DHS program is granted through the U.S Department of Health and Human Services, and in addition, the Nigerian DHS is conducted according to local Nigerian research ethics requirements. Data for this analysis were accessed via the publicly available DHS datasets, with access granted by the DHS program. As this was a secondary data analysis, further research ethics approval was not required, however, in accordance with DHS regulations, all data extracted from the NDHS for the purpose of this study were handled as confidential and survey respondents remained unidentified. This study conforms to the principles of the Declaration of Helsinki.

## Results

### Sample characteristics

In the 2003 sample, 36.6% had less than primary education, 48.4% were literate, 58.6% employed, and 64.8% rural-dwelling, whilst in the 2008 sample 30.8% of respondents had less than primary education, 53.6% were literate, 65.6% were employed, and 63.7% resided in rural areas. Lastly, 32.7% of the 2013 sample had less than a primary school education, 52.9% were literate, 66.6% employed, and 57.3% rural-dwelling. Further socio-demographic characteristics are shown in Table 1**.**

**Table 1:** Demographic Characteristics of Nigerian Demographic and Health Survey Respondents in 2003, 2008 and 2013

Demographic characteristics		Year					
		2003		2008		2013	
		Number	Percent	Number	Percent	Number	Percent
Sex	Male	2093	21.6	13,808	29.3	17,359	30.8
	Female	7620	78.4	33,385	70.7	38,948	69.2
Age Group (Years)	15-24	4089	42.1	17,537	37.2	21,088	37.5
	25-34	2950	30.4	15,459	32.8	17,783	31.6
	35-49	2674	27.5	14,197	30.1	17,436	31.0
Rural / Urban Residence	Urban	3421	35.2	17,150	36.3	24,026	42.7
	Rural	6292	64.8	30,043	63.7	32,281	57.3
Ethnicity	Other	3631	37.4	15,008	31.9	19,225	34.1
	Fulani	576	5.9	2764	5.9	3518	6.2
	Hausa	2584	26.6	10,538	22.4	15,417	27.4
	Ibibio	353	3.6	1159	2.5	1261	2.2
	Igbo	1323	13.6	7294	15.5	7967	14.1
	Ijaw	118	1.2	1790	3.8	1097	1.9
	Yoruba	1127	11.6	8479	18.0	7823	13.9
Religion	Other	12	0.1	112	0.2	298	0.5
	Catholic	1307	13.5	5444	11.6	6329	11.2
	Other Christian	3375	34.8	19,866	42.3	20,102	35.7
	Islam	4910	50.6	20,999	44.7	29,057	51.6
	Traditionalist	103	1.1	566	1.2	521	0.9
Relationship Status	Never married	2974	30.6	14,945	31.7	17,704	31.4
	Currently married	6342	65.3	30,596	64.8	36,552	64.9
	Formerly married	398	4.1	1647	3.5	2051	3.6
Highest Educational Attainment	No education	3555	36.6	14,539	30.8	18,414	32.7
	Primary	2148	22.1	9327	19.8	9640	17.1
	Secondary	3303	34.0	18,374	38.9	22,208	39.4
	Higher	707	7.3	4953	10.5	6044	10.7
Literacy Level	Low literacy/ illiterate^a^	4913	51.6	21,697	46.4	26,354	47.1
	Literate	4615	48.4	25,073	53.6	29,542	52.9
Employment Status	Unemployed	4011	41.4	16,132	34.4	18,720	33.4
	Employed	5681	58.6	30,793	65.6	37,305	66.6
National Wealth Quintile	Lower 20th	1776	18.3	8469	17.9	9994	17.7
	40th	1799	18.5	8566	18.2	10,420	18.5
	60th	1905	19.6	8910	18.9	10,824	19.2
	80th	1978	20.4	10,101	21.4	11,827	21.0
	Upper 20th	2255	23.2	11,147	23.6	13,242	23.5
State-level Wealth Inequality Ratio	< 1.50	449	4.6	3646	7.7	8012	14.2
	1.50-1.99	921	9.5	3770	8.0	33,742	59.9
	2.00-2.49	754	7.8	9800	20.8	12,464	22.1
	2.50-3.00	2021	20.8	10,807	22.9	2088	3.7
	3.01-4.00	3097	31.9	9690	20.5	0	0.0
	4.01-5.00	1419	14.6	7448	15.8	0	0.0
	> 5.00	1052	10.8	2031	4.3	0	0.0

^a^Includes the visually impaired

### Trends in HIV-related knowledge, 2003-2013

The overall HIV-related knowledge score for the samples [mean (SD)] were 9.18 (1.83), 8.09 (2.80) and 8.62 (2.50) in 2003, 2008, and 2013, respectively. Mean scores for the subdomains of HIV-related knowledge in each year are shown in Table 2.

**Table 2:** Mean HIV-Related Knowledge Scores in the Nigerian Population, 2003-2013

HIV-Related Knowledge Score	Year					
	2003		2008		2013	
	Mean	SD	Mean	SD	Mean	SD
General HIV-Related Knowledge	2.09	0.77	2.37	0.71	2.43	0.68
Mother-to-Child-Transmission	2.69	0.67	1.70	1.31	1.84	1.24
Other Modes of Transmission	1.84	1.05	2.02	1.06	2.22	0.98
HIV Risk Reduction	2.02	0.84	1.99	0.98	2.13	0.95
Total, Overall HIV-Related Knowledge	9.18	1.83	8.09	2.80	8.62	2.50

Kruskal-Wallis tests indicated that the distribution of HIV-related knowledge scores differed significantly across years for all knowledge domains, as well as for overall HIV-related knowledge (all *p* < 0.001).

**Fig. 1:**
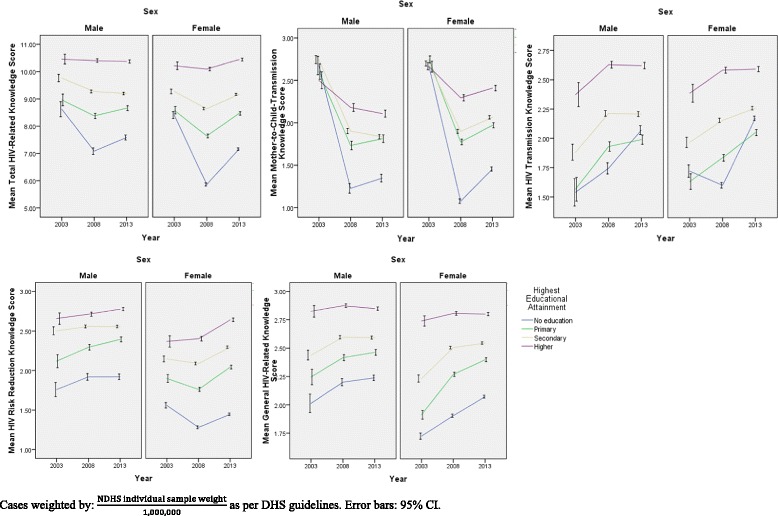
HIV-Related Knowledge in Nigeria by Sex and Educational Attainment, 2003-2013

**Fig. 2:**
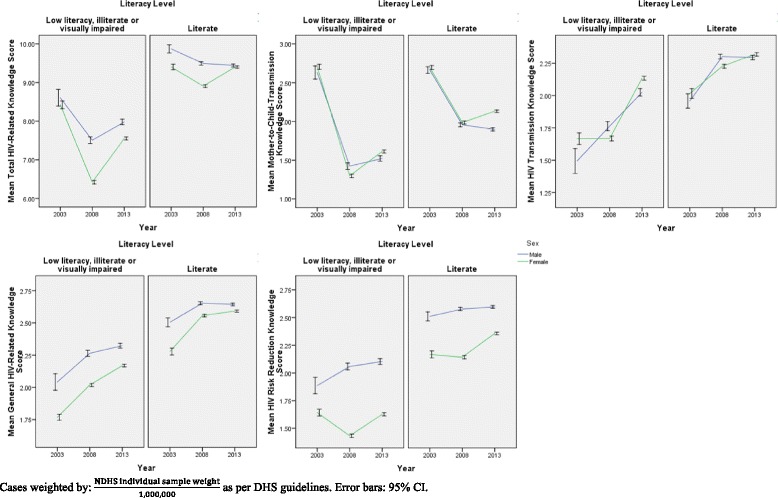
HIV-Related Knowledge in Nigeria by Sex and Literacy Level, 2003-2013

**Fig. 3:**
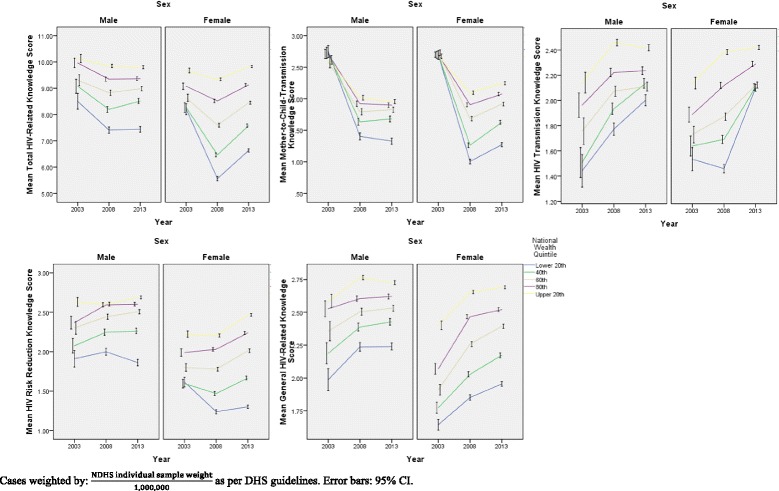
HIV-Related Knowledge in Nigeria by Sex and National Wealth Quintile, 2003-2013

**Fig. 4:**
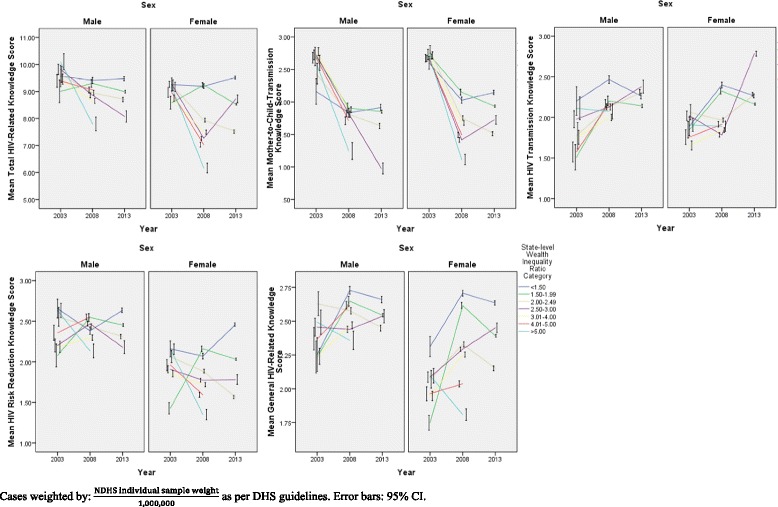
HIV-Related Knowledge in Nigeria by Sex and Wealth Inequality Ratio Category, 2003-2013

Across all HIV-related knowledge domains apart from general HIV-related knowledge, scores were generally found to be lower in 2008 than in 2003 or 2013. Furthermore, alarmingly, although knowledge levels for the other knowledge subdomains were higher in 2013 than in 2003, knowledge of mother-to-child transmission (MTCT) dropped significantly in 2013 in comparison to 2003 levels (*p* < 0.001, as derived from Kruskal-Wallis analysis). This trend in mean HIV-related knowledge domains over the 2003-2013 period, stratified by various socio-demographic variables, is shown in Figs. 1, 2, 3 and 4**.**

It is, in addition, interesting to note that in general, across time points, HIV-related knowledge is higher in males than in females for all knowledge domains, except knowledge of mother-to-child transmission (MTCT). Moreover, HIV-related knowledge is higher at each higher level of wealth, as well as being higher for literate compared to illiterate respondents, and these observations remain true over time. Moreover, as seen in Fig. 3, particularly in the risk reduction knowledge panel, although knowledge levels are generally similar among males and females when wealth inequality is low (wealth inequality ratio < 1.50), the decrease in risk reduction knowledge is more pronounced among females than among males at higher levels of wealth inequality.

**Fig. 5:**
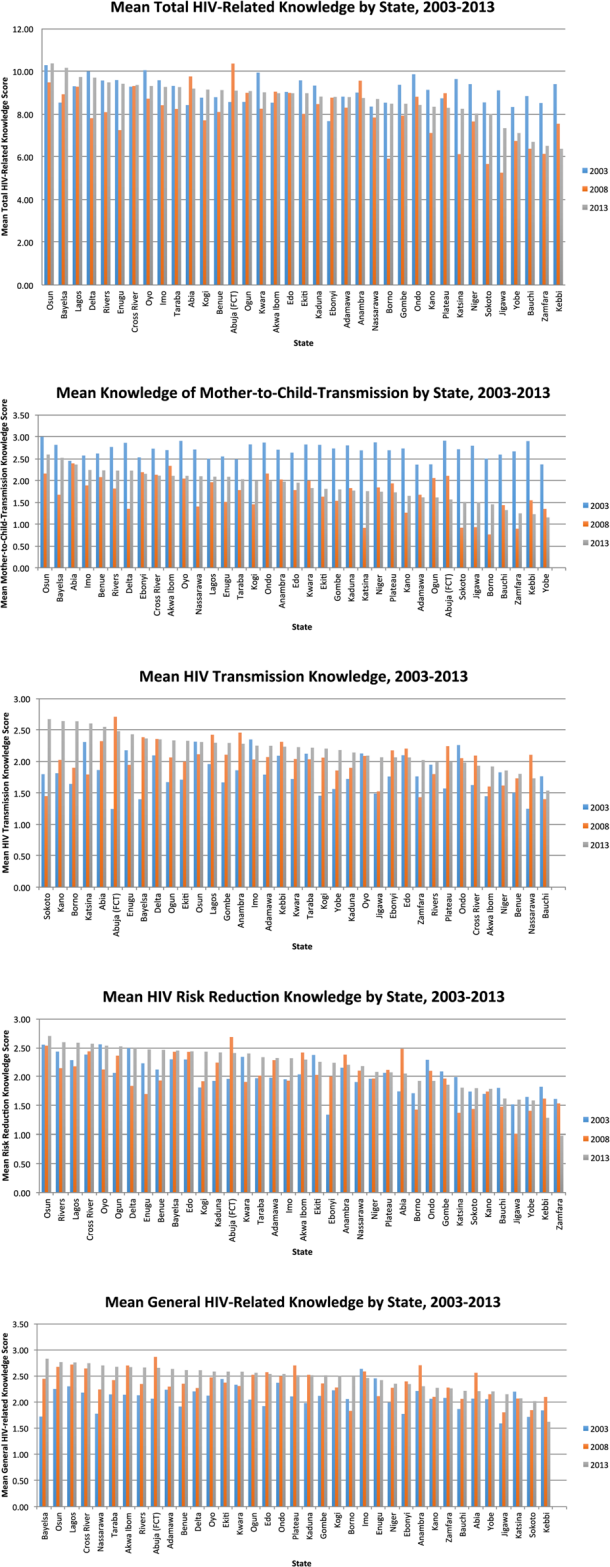
HIV-Related Knowledge by State in Nigeria, 2003-2013

Lastly, mean HIV-related knowledge scores in each state from 2003 to 2013 are shown in Fig. 5. States with relatively low scores across all knowledge domains as of 2013 include states such as Zamfara, Yebbi and Bauchi in the north of the country, whilst states further south, such as Osun and Bayelsa, had higher 2013 HIV-related knowledge scores across most knowledge domains. It is notable that in some knowledge domains, the disparity between HIV-related knowledge between states is large. For example, the mean score for risk reduction knowledge in 2013 ranges from 2.70 in Osun to only 0.98 in Zamfara, and this disparity has grown larger over time, being greater in 2013 than in the previous years.

**Fig. 6:**
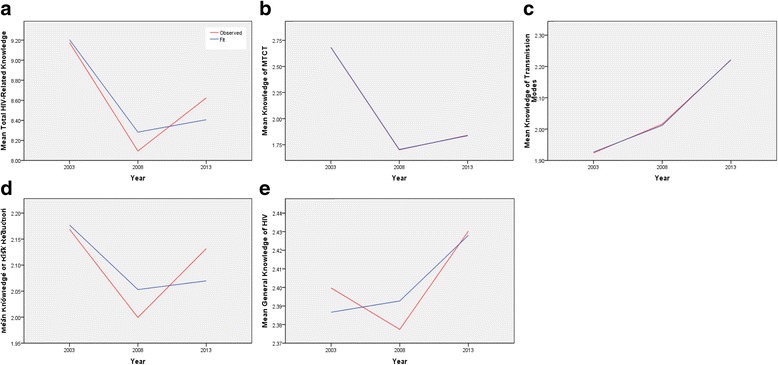
2003-2013 Time Series Analysis: ARIMA Models for Total HIV-related Knowledge and Knowledge Subdomains in the Nigerian Population. Predictors included in ARIMA model: **a**, **b**, **d** Proportion of literate respondents. **c** Mean state-level wealth inequality ratio. **e** Proportion of respondents living in rural areas

In time series analyses of total HIV-related knowledge, an ARIMA model with the proportion of literate respondents in each year as a single predictor yielded the highest R-squared value (0.857), as was also the case for the MTCT and risk reduction knowledge domains (R-squared values 1.000 and 0.572, respectively). For knowledge of HIV transmission, the best model fit was obtained with wealth inequality as single predictor (R-squared 0.999), whilst for general HIV-related knowledge, the proportion of rural-dwelling respondents in each year as predictor provided the best fit (R-squared 0.709). The ARIMA models for total HIV-related knowledge and each knowledge domain over the 2003 to 2013 time period are shown in Fig. 6. Forecasts could not be generated by any of the models due to data limitations.

## Discussion

When considering the implications of an analysis of trends in HIV-related knowledge, it is relevant to note at the outset that several Sub-Saharan African studies have reported the effectiveness of behaviour change interventions regarding HIV risk reduction and prevention measures [20–22]. This underlines firstly that interventions improving HIV-related knowledge, when delivered along with behaviour change elements, are associated with a greater likelihood of adoption of appropriate preventive behaviours, and in turn therefore a reduced risk of HIV transmission [22], and secondly that HIV-related knowledge is a modifiable factor that can and should be targeted through intervention, as part of efforts to prevent further HIV transmission. Regarding the socio-demographic determinants of HIV-related knowledge in Nigeria, a recent paper has shown that factors including poverty, unemployment, low literacy, rural residence, sex, and wealth inequality are significant predictors of HIV-related knowledge in the country [12], With this in mind, the implications of the current trend analysis in the Nigerian context are discussed.

The low level of HIV-related knowledge seen in 2008 in the current analysis may be explained in part by the fact that HIV-related knowledge in Nigeria may have been low prior to the launching of the National Strategic Plan to combat HIV, which was initiated in 2010. The subsequent rise in 2013 suggests that the plan may have to some extent been effective in increasing overall HIV-related knowledge at the national level, however, due to the limited amount of data, the influence of other societal dynamics and events on this trend remains to be better understood.

Despite this decrease observed in most knowledge domains in 2008, an overall increase is seen from 2003 to 2013 in all knowledge domains except knowledge of mother-to-child transmission, which was significantly lower in 2013 than in 2003. This is particularly alarming given the substantial contribution of MTCT to the continuation of the Nigerian HIV epidemic, with a MTCT prevalence rate of 27.3% in 2014 [10], This suggests that future national programs with an HIV prevention and education mandate should place an increased focus on the prevention of MTCT. Specific recommendations regarding MTCT prevention based on the findings of the current study include that educational interventions emphasizing safe infant feeding practices and encouraging antenatal and postnatal care seeking should be targeted at expectant mothers and females of childbearing age, in particular those who are HIV positive or whose serostatus is unknown. In addition, as mother-to-child transmission knowledge is the only knowledge area in which males scored lower than women, it may be of interest to also include mother-to-child transmission prevention content in HIV education efforts targeted at males. Given that males may often be the primary household decision-makers, they may be more likely to support their partners in seeking maternal care or in making alternative infant feeding choices if adequately informed of the risks of mother-to-child transmission.

The overall increase in the other HIV-related knowledge domains over time seen in our analysis however aligns with the findings of studies that have examined trends in HIV-related knowledge in other sub-Saharan African countries. The aforementioned study in Ethiopia for example reports an increase in HIV-related knowledge between 2005 and 2011 [8]. As knowledge levels in the Ethiopian study were based on only 3 knowledge indicators, one being having heard of HIV, and two relating to risk reduction measures, these results are comparable to the upward trend in the general knowledge and HIV risk reduction domains observed in the current study.

Importantly, although the trends in HIV-related knowledge are similar across socio-demographic strata, there are marked disparities in the levels of knowledge between strata. For example, females generally have lower HIV-related knowledge across most knowledge domains in comparison to males, and those in higher wealth quintiles have higher mean HIV-related knowledge levels than those in lower wealth quintiles. This, as well as the disparities seen in levels of HIV-related knowledge among males compared to females at each level of wealth, and in particular the fact that mean risk reduction knowledge is similar among males and females at low wealth inequality, but is much lower in females than in males in states with higher wealth inequality ratios, suggests that future HIV awareness and education campaigns should be targeted at the most marginalized, particularly those experiencing the confluence of gender and wealth inequalities.

Moreover, the fact that disparities in HIV-related knowledge between literate and illiterate respondents persist from 2003 to 2013 across all knowledge domains suggests an urgent need for the tailoring of future national HIV education programs to the needs of those to whom complex or text-based information is less accessible. This should include the use of non-written media (for example diagrams or pictograms) for the dissemination of HIV-related information, including transmission mechanisms and prevention measures.

Furthermore, the results of this analysis regarding the observation of growing disparities in certain domains of HIV-related knowledge between states suggest that increased focus should be devoted to improving HIV-related knowledge in these specific states in which it is currently low, such as in Zamfara, Kebbi, and Bauchi. In particular, efforts to improve HIV-related knowledge in these states should focus on the specific knowledge subdomains that are currently most poorly understood.

Regarding the trend analysis using ARIMA modelling, the fact that the proportion of literate respondents as a predictor provided the best fit model for total HIV-related knowledge, risk reduction knowledge, and knowledge of mother-to-child transmission suggests that at the national level, improvements in literacy over time may in part explain and facilitate improvements in HIV-related knowledge. Consequently, this indicates that not only should efforts be made to ensure that HIV education campaigns are more accessible to individuals with low literacy levels, but also that investments into national education and literacy in general will equip individuals with a greater capacity to acquire, understand, and use HIV-related information.

Moreover, the finding that knowledge of HIV transmission in Nigeria was best approximated in the ARIMA model using the mean state-level wealth inequality ratio as the single predictor is of particular interest in light of recent studies on the social determinants of HIV transmission indicating that, in Sub-Saharan Africa, wealth inequality may be a more significant predictor of HIV transmission than absolute poverty or wealth [13–16]. Additionally, in Nigeria specifically, a recent study investigating wealth inequality as a predictor of HIV-related knowledge [12] indicated that under circumstances of inequality, females in particular are at higher risk of low HIV-related knowledge. The observation that the trend in knowledge of HIV transmission to some extent follows the trend in state-level wealth inequality therefore underlines that under circumstances of inequality, individuals experience both greater barriers to accessing HIV-related health information, as well as greater barriers to the actual application of this information through the adoption of preventive or care-seeking behaviours. Apart from indicating that, particularly in a country as socioeconomically heterogeneous as Nigeria, HIV prevention education should be especially targeted at areas of high wealth inequality, the observed HIV transmission knowledge and wealth inequality trends also suggest that efforts towards reducing wealth disparities in Nigeria could address an important driver of HIV transmission in the country, and consequently substantially reduce future transmission. More specifically, as the current study shows a more pronounced decrease in risk reduction knowledge among females than among males at higher levels of wealth inequality, females living in areas of high wealth inequality should be particularly prioritized for HIV risk reduction interventions. This is particularly relevant given that circumstances of wealth inequality have been shown to be associated with an increase in high HIV risk activities, such as engagement in informal transactional sex [23, 24].

The limitations of this study include, firstly, the limited number of time points in this analysis, which limited the time series analysis as it precluded the production of trend forecasts from the ARIMA models, therefore limiting conclusions regarding future HIV-related knowledge levels in the country. In addition, the small number of time points between 2003 and 2013 limits the level of detail in our understanding of the trends in HIV-related knowledge over time in the Nigerian context, making it difficult to interpret what these knowledge levels suggest regarding the effectiveness of HIV awareness and education campaigns implemented over the years, or to determine which other events or dynamics may be contributing to the observed trends in knowledge levels.

In addition, the fact that NDHS data is not longitudinal – i.e. not collected from the same individuals over the multiple time points – means that longitudinal data analysis methods are not applicable, and conclusions from this analysis are thus unable to take into account how individual-level changes in absolute wealth, wealth inequality, educational attainment or other socio-economic indicators influence changes in individual-level HIV-related knowledge over time.

It should also be noted that as the DHS sampling procedure includes women in all sampled households and the corresponding men in only a subset of the households from the original female sample, there is a greater representation of women than men in all survey years, however, the effects of this are taken into account through the application of individual sample weights (as provided in the DHS) to the male and female datasets.

Lastly, although health-related knowledge has been shown to lead to favourable health behaviours and engagement in preventive measures and can therefore be considered a relevant factor influencing potential HIV infection risk, the lack of individual HIV serostatus information in the NDHS limits our ability to corroborate the contribution of low HIV-related knowledge to HIV transmission risk. Consequently, the conclusions drawn from an analysis of HIV-related knowledge are of limited value in terms of their direct translation into the evidence-based targeting of HIV preventive interventions among high-risk groups. Future research in Nigeria could therefore focus on the collection of individual-level HIV serostatus data for the determination of whether HIV-related knowledge is a valuable predictor of HIV transmission risk. Moreover, more detailed evaluations of national HIV education and prevention programs in terms of their effectiveness in disseminating HIV preventive information to vulnerable groups, improving HIV-related knowledge, and ultimately leading to preventive behaviours are needed.

## Conclusions

Overall, through the identification of states, population subgroups and knowledge domains (such as knowledge of MTCT) in which HIV-related knowledge levels have been low or decreasing over time, the understanding of recent trends in HIV-related knowledge as investigated in this paper provides insight for the evidence-based design and targeting of further HIV education efforts. In light of the current study’s findings, we suggest several specific strategies for HIV-related knowledge improvement in Nigeria, relating to both the content and method of dissemination of HIV education interventions. Taking continued steps towards improving HIV-related knowledge in Nigeria is particularly relevant in light of the fact that the aforementioned study on attitudes towards HIV in Nigeria demonstrated that individuals with greater understanding of HIV transmission were also less likely to have negative or stigmatizing attitudes towards people living with HIV [11]. This indicates that appropriate HIV education and subsequent improvements in HIV-related knowledge are crucial to reducing the social stigmatization of the disease, which consequently will contribute to increasing at-risk individual’s propensity and ability to seek preventive services, as well as facilitating easier access to treatment and strengthening social support for HIV-positive individuals. In addition however, as a prior Sub-Saharan African study has noted discrepancies between HIV awareness and the adoption of protective behaviours [25], it will be crucial that future HIV education efforts also incorporate elements of behaviour change interventions, in order to maximize their likelihood of resulting in actual increases in preventive behaviours.

## Appendix

**Table 3:** Questions included in the computation of the HIV-related knowledge scores in the current study

Question	Coding	
	Yes	No
1. Has heard of AIDS	1	0
2. Knows a place to get HIV testing	1	0
3. Knows a source for condoms^a^	1	0
4. To reduce the risk of getting HIV: always use condoms during sex	1	0
5. To reduce the risk of getting HIV: have one sex partner only, who has no other partners	1	0
6. Can contract HIV from mosquito bite	0	1
7. Can contract HIV by sharing food with person who has AIDS	0	1
8. Can contract HIV by witchcraft or supernatural means	0	1
9. A healthy looking person can have HIV	1	0
10. HIV can be transmitted during pregnancy	1	0
11. HIV can be transmitted during delivery	1	0
12. HIV can be transmitted by breastfeeding	1	0

^a^Initially “does not know any source of condoms” in NDHS, re-defined as “knows a source of condoms” for ease of interpretation

## Abbreviations

AIDS: Acquired Immunodeficiency Syndrome
ANOVA: Analysis of Variance
ARIMA: Autoregressive Integrated Moving Average
DHS: Demographic and Health Survey
HIV: Human Immunodeficiency Virus
MAPE: Mean Absolute Percentage Error
MTCT: Mother-to-Child Transmission
NDHS: Nigerian Demographic and Health Survey
NSP: National Strategic Plan to Combat HIV/AIDS
SD: Standard Deviation
